# Synergistic antitumor effects of 9.2.27-PE38KDEL and ABT-737 in primary and metastatic brain tumors

**DOI:** 10.1371/journal.pone.0210608

**Published:** 2019-01-09

**Authors:** Xin Yu, Mikhail Dobrikov, Stephen T. Keir, Matthias Gromeier, Ira H. Pastan, Ralph Reisfeld, Darell D. Bigner, Vidyalakshmi Chandramohan

**Affiliations:** 1 Department of Pathology, Duke University Medical Center, Durham, NC, United States of America; 2 Department of Neurosurgery, Duke University Medical Center, Durham, NC, United States of America; 3 Laboratory of Molecular Biology, Center for Cancer Research, National Cancer Institute, National Institutes of Health, Bethesda, MD, United States of America; 4 Department of Immunology and Microbiology, The Scripps Institute, La Jolla, CA, United States of America; University of South Alabama Mitchell Cancer Institute, UNITED STATES

## Abstract

Standard treatment, unfortunately, yields a poor prognosis for patients with primary or metastatic cancers in the central nervous system, indicating a necessity for novel therapeutic agents. Immunotoxins (ITs) are a class of promising therapeutic candidates produced by fusing antibody fragments with toxin moieties. In this study, we investigated if inherent resistance to IT cytotoxicity can be overcome by rational combination with pro-apoptotic enhancers. Therefore, we combined ITs (9.2.27-PE38KDEL or Mel-14-PE38KDEL) targeting chondroitin sulfate proteoglycan 4 (CSPG4) with a panel of Bcl-2 family inhibitors (ABT-737, ABT-263, ABT-199 [Venetoclax], A-1155463, and S63845) against patient-derived glioblastoma, melanoma, and breast cancer cells/cell lines. *In vitro* cytotoxicity assays demonstrated that the addition of the ABT compounds, specifically ABT-737, sensitized the different tumors to IT treatment, and improved the IC_50_ values of 9.2.27-PE38KDEL up to >1,000-fold. Mechanistic studies using 9.2.27-PE38KDEL and ABT-737 revealed that increased levels of intracellular IT, processed (active) exotoxin, and PARP cleavage correlated with the enhanced sensitivity to the combination treatment. Furthermore, we confirmed the synergistic effect of 9.2.27-PE38KDEL and ABT-737 combination therapy in orthotopic GBM xenograft and cerebral melanoma metastasis models in nude mice. Our study defines strategies for overcoming IT resistance and enhancing specific antitumor cytotoxicity in primary and metastatic brain tumors.

## Introduction

Glioblastoma (GBM), arising from glial cells, is the most frequent and most malignant primary brain tumor in adults. The median survival (MS) for newly diagnosed GBM patients treated with the current standard of care, including surgery, radiation, and temozolomide chemotherapy, is 15 to 18 months [1, 2]. Conversely, brain metastases occur in 5–7% of patients with melanoma and breast cancer [3]. The MS for melanoma and breast cancer patients with brain metastases with the current standard of care, including surgery, radiation, and systemic immunotherapy or chemotherapy is 29 and 2 to 25 months, respectively [4, 5]. These poor outcomes mandate a need for the development of improved therapeutic options.

Tumor-targeted therapy is highly desirable due to its high specificity and potency in multiple cancer types [6–8]. Among the targeted therapies under development, immunotoxins (ITs) have emerged as a class of promising therapeutic candidates [9]. ITs are produced by genetically fusing single-chain variable-region antibody fragments (scFvs) to a toxin molecule, such as the 38 kDa truncated mutant form of *Pseudomonas* exotoxin A (PE38) [10]. An improved PE38 variant (PE38KDEL), was designed with a C-terminal KDEL addition to increase the intracellular retention and cytotoxicity of the ITs [11, 12]. ITs bind to cell surface antigens via the scFv portion. Upon antigen binding, they are internalized into endosomes, and the PE38KDEL moiety is cleaved by furin. The catalytically active C-terminal fragment then translocates to the cytosol via the endoplasmic reticulum (ER), where it inactivates elongation factor 2 (EF2) by ADP-ribosylation of the EF2 diphthamide residue, leading to protein synthesis inhibition and apoptosis [11].

The utility and specificity of ITs are dictated by the targeted tumor antigen. Numerous cell-surface tumor antigens have been investigated as therapeutic targets in brain tumors. The melanoma- and glioma-associated antigen chondroitin sulfate proteoglycan 4 (CSPG4) is overexpressed in 90% of melanomas and gliomas as well as in breast cancer [13–15]. CSPG4 is expressed on ‘cancer stem-like cell populations’ and promotes radiation resistance in GBM [16]. CSPG4 is recognized by monoclonal antibodies (mAbs) Mel-14 [13] and 9.2.27 [17]. Mel-14 and 9.2.27 mAbs target different regions of the extracellular domain on CSPG4 and the anti-tumor efficacy of one mAb over the other is unknown. To determine the utility of Mel-14 and 9.2.27 mAbs for targeted brain tumor therapy, we developed two CSPG4-targeting scFv based immunotoxins, Mel-14-PE38KDEL and 9.2.27-PE38KDEL.

Compared to other targeted therapeutic approaches, ITs are attractive due to their excellent safety profile and potent cytotoxicity in the pM-fM range [11, 12, 18, 19]. Despite these advantages, cancer cells usually do not exhibit homogeneous susceptibility to ITs. Small molecule Bcl-2 inhibitors (ABT-737, ABT-263, and ABT-199 [Venetoclax]) [20, 21] were proposed to combat inherent tumor cell resistance to ITs by studies in cervical adenocarcinoma, pancreatic cancer, and small cell lung cancer [22–24]. ABT-737, along with its analogs ABT-263 and ABT-199, are BH-3 mimetics that target anti-apoptotic pathways by binding to and neutralizing the pro-survival members of the Bcl-2 family proteins, such as Bcl-2, Bcl-xL, and Bcl-w, thereby promoting apoptosis via the release of Bax and Bak proteins [25–27].

In this study, we evaluated the synergistic effect of ITs (Mel-14-PE38KDEL or 9.2.27-PE38KDEL) targeting CSPG4 and one of the five small molecule Bcl-2 family inhibitors, ABT-737 and ABT-263 (specific for Bcl-2, Bcl-xL, and Bcl-w), ABT-199 (specific for Bcl-2), A-1155463 (specific for Bcl-xL) [28], or S63845 (specific for Mcl-1) [29, 30] in GBM xenograft cells, and melanoma and breast cancer cell lines. We determined the *in vitro* cytotoxicity of the Bcl-2 family inhibitors/IT monotherapies and combination therapies using a colorimetric cell proliferation assay (WST-1). Further, we established the *in vivo* efficacy of the ABT-737 and 9.2.27-PE38KDEL combination in intracranial GBM patient-derived xenograft (PDX) and melanoma brain metastasis models. Mechanistic studies revealed the factors contributing to the efficacy of ABT-737/9.2.27-PE38KDEL combination therapy in GBM, melanoma, and breast cancer cells.

## Materials and methods

### Xenografts and cell lines

Human GBM xenografts (D-10-0021 MG, D-245 MG, and D-08-0326 MG) were established from patient tumors obtained through informed consent. The subcutaneous xenografts were harvested when the tumors reached an average volume of 500 mm^3^ to 750 mm^3^; tumor harvest date was between 30–45 days post-implantation. None of the subcutaneous tumors succumbed to ulceration or blistering. The maximum tumor volume threshold for the subcutaneous xenografts was set at 1500 mm^3^ per our approved animal protocol A049-17-02.

Human melanoma cell line (H350) were maintained in our laboratory. Human melanoma cell lines (DM440 and DM443) [31] were kindly provided by Dr. Douglas S. Tyler at Duke University. Human breast cancer cell lines (SUM159, SUM159-R113, and Hs 578T) [32] were kindly provided by Dr. Robin Bachelder at Duke University. All cells were cultured in an incubator at 37°C, 5% CO_2_, and passaged when reached confluence with Accutase Cell Detachment Solution (BD Biosciences #561527). All GBM xenografts and the melanoma cell line H350 were maintained in 1x MEM ZINC Option media (Gibco #05-0009DJ) supplied with 10% fetal bovine serum (FBS). DM440 and DM443 cells were cultured in DMEM media (Gibco #11995) supplied with 10% FBS. SUM159 and SUM159-R113 cells were grown in Ham’s F-12 base media (Gibco #11765) with the addition of 5% FBS, 1 μg/ml hydrocortisone, and 5 μg/ml human insulin. Hs 578T cells were maintained in DMEM media (Gibco #11995) with the addition of 10% FBS and 10 μg/ml human insulin.

### Western analysis

#### Preparation of total cell lysates

Cells were harvested and solubilized in polysomal lysis buffer (100 mM KCl, 5 mM MgCl_2_, 10 mM HEPES pH7.0, and 0.5% NP-40) supplemented with Halt Protease & Phosphatase inhibitor cocktail (Thermo Scientific #78440). Protein concentrations were determined using the Bradford protein assay (Bio-Rad #5000001). Equal amounts (5–10 μg) of total protein were loaded onto NuPAGE 4%-12% Bis-Tris gels (Invitrogen #NP0321) and transferred to nitrocellulose membranes (GE #10600000) for staining.

#### Preparation of cell fractionations

Cells were harvested and resuspended in a cytosol extraction buffer [110 mM KOAc, 25 mM K-HEPES (pH 7.5), 25 μM MgCl_2_] supplemented with 1 mM DDT, 150 μg/ml digitonin (Sigma #D141) and protease inhibitor cocktail. Cells were incubated on ice for 15 min, then the supernatant containing the cytosolic fraction was harvested by centrifugation at 10000 g for 10 min. The permeabilized cells were washed and centrifuged at 500 g for 2 min at 4°C. The pellets were resuspended in an ER-solubilization buffer [200 mM KCl, 25 mM K-HEPES (pH 7.5), 10 mM MgCl2] supplemented with 1 mM DDT, 20 mg/ml n-dodecyl-b-D- maltoside (Sigma # D4641) and protease inhibitor cocktail. After a 30-min incubation on ice, the mixture was centrifuged at 10000 g for 10 min to remove nuclei, mitochondria, and incompletely solubilized cellular debris.

#### Global translation inhibition assay

Cells were treated with 5 μM puromycin (Tocris #4089) for 15 min, then washed twice and immediately lysed in polysomal lysis buffer supplemented with protease inhibitor cocktail.

#### Drugs and antibodies

ABT-737 was prepared using the same method as described in the WST-1 assay. Primary antibodies against the following targets were purchased from Cell Signaling Technology: p-FAK(#8556), FAK (#7143), p-AKT (#2965), AKT (#9272), p-PKCα (#9375), PKCα (#2056), GAPDH (#2118), Bcl-2 (#4223), Bcl-xL (#2764), Mcl-1 (#5453), Bim (#2933), Bax (#5023), and PARP (#9542). Antibodies against puromycin (Millipore #MABE343) and furin (ProteinTech #18413-1-AP) were also obtained. An anti-PE38 antibody capable of binding to both intact and cleaved PE38 toxins was kindly provided by Dr. Ira Pastan’s laboratory at National Institutes of Health (NIH). All experiments were repeated at least 2 times.

### Animal work approval

All animal work described in the manuscript have been reviewed and approved by the following animal research ethics committee of Duke University Medical Center: Duke Occupational and Environmental Safety Office, Duke Office of Animal Welfare Assurance, Duke Veterinary Committee, and finally the Duke Institute of Animal Care and Use Committee. The protocol number for the approved animal work is A049-17-02.

### Anesthesia

Isoflurane anesthesia (Absolute Anesthesia Inc.) via inhalation was used during surgical procedures as it stabilizes the mice during surgery and allows for efficient post-surgical recovery.

### Euthanasia

CO_2_, inhalation followed by decapitation was used for euthanasia. All animals were euthanized in accordance with the established humane endpoints listed in our approved animal protocol A049-17-02. For subcutaneous tumors, the humane endpoint is reached when tumor burden reaches 1500 mm^3^. For intracranial tumors, the humane endpoint is reached using neurological symptoms and body weight loss of 15% or the animals’ inability/desire to move (the mouse does not move forward two steps when prompted gently).

### Intracranial tumor models

All experiments were done in accordance with the Institutional Animal Care and Use Committee of Duke University Medical Center (A049-17-02). Animals were group-housed, maintained in a barrier facility, under pathogen-free conditions according to NIH guidelines. Nude mice (≈22–30 g, 6–8 weeks, female:male = 1:1, Duke University, Division of Laboratory Animal Resources) were anesthetized by isoflurane inhalation and mounted onto the stereotactic frame (Stoelting Co.). The anterior cranial region was shaved, and an incision ≈1 cm in length was made in the skin over the skull. A 25-gauge needle attached to a 25-μl Hamilton syringe was used to pierce through the skull at coordinates 2.0 mm left lateral of the sagittal and 0.5 mm anterior to the bregma. The needle was inserted vertically to a depth of 2.5 mm from the dura mater. A total of 1x10^5^ D-10-0021 MG GBM cells freshly dissociated from a subcutaneous mouse xenograft, or 1x10^5^ DM440 melanoma cells harvested from culture, were injected in 5 μl of 1xPBS containing 2% methylcellulose (Sigma #M0512).

Five days post-tumor implantation, the animals were randomized into four treatment groups, with 9–10 mice per group: vehicle control, 9.2.27-PE38KDEL, ABT-737, and 9.2.27-PE38KDEL/ABT-737 combination therapy, randomized according to initial weight. For each mouse in the study, a brain infusion cannula (Alzet #0008851) attached to a subcutaneously implanted mini-osmotic pump (Alzet #1007D) was inserted directly into the intracranial tumor site for intratumoral delivery of the vehicle solution or the drugs, at a rate of 0.5 μl/h for 72 hours. The vehicle control group received the PBS-based solution containing 5% Captisol and 2% mouse serum albumin. The IT monotherapy group received a total dose of 0.1 μg of 9.2.27-PE38KDEL diluted in the vehicle solution. The ABT-737 group received a total dose of 2.93 μg of ABT-737 (equivalent to 36 μl of the 100 μM of ABT-737 diluted in the vehicle control solution). The combination group received both 9.2.27-PE38KDEL and ABT-737 at the doses indicated above. During the surgery, animals in different groups were treated in a random order. Animals were observed twice daily for signs of distress. Survival endpoint is defined as the onset of neurologic symptoms (lethargy, seizure, repetitive circling, difficulty breathing, and hunched posture), greater than 15% loss of body weight, or death, whichever comes first. When endpoint was reached, mice were euthanized as described earlier. Kaplan-Meier survival curves were plotted and compared using log-rank test. Each study was repeated at least twice.

## Results

### Expression of CSPG4 on tumor cells and *in vitro* cytotoxicity of IT, ABT, A-1155463, and S63845 monotherapies

**Fig 1:**
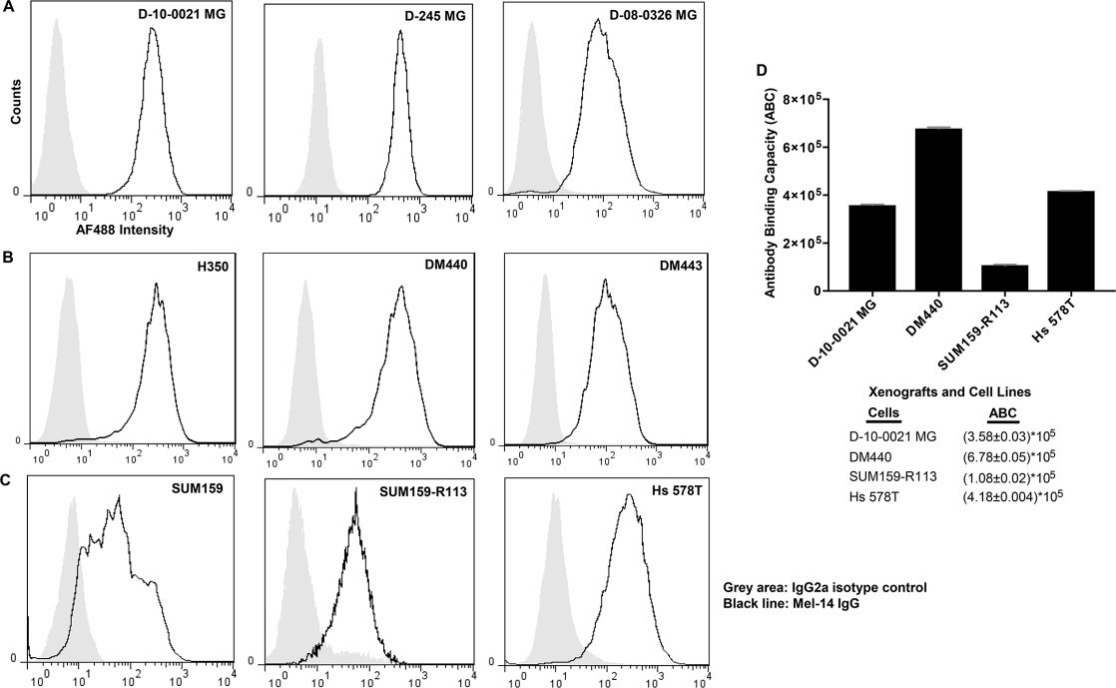
CSPG-4 expression in human tumors. **A-C.** Flow cytometry analysis of CSPG-4 expression on GBM xenografts (A), melanoma cell lines (B), and breast cancer cell lines (C). **D.** Quantitative flow cytometry (Q-FACS) analysis of CSPG-4 binding capacity on selected xenografts and cell lines. The values represent the average of 2 experiments, and the error bars represent SEM.

Flow cytometry analysis (FACS) revealed the presence of CSPG4 on all GBM, breast cancer, and melanoma cell lines tested, albeit at varying levels (Fig 1A–1C). Q-FACS analysis showed that the surface density of CSPG4 was lowest in SUM159-R113 cells (≈100,000 molecules per cell) and highest in DM440 cells (≈700,000 molecules per cell) (Fig 1D). Despite abundant CSPG4 cell surface expression, *in vitro* cytotoxicity assays demonstrated that all GBM xenografts, all breast cancer cell lines, and the DM443 melanoma cell line were highly resistant to both Mel-14-PE38KDEL and 9.2.27-PE38KDEL ITs (IC_50_ >100 ng/ml) (Tables 1 and 2). Only the H350 melanoma cells were susceptible to the cytotoxicity of 9.2.27-PE38KDEL (IC_50_ of 11.67 ng/ml; Table 2). Both H350 (IC_50_ = 42.50 ng/ml) and DM440 (IC_50_ = 68.30 ng/ml) melanoma cells showed weak cytotoxicity upon Mel14-PE38KDEL treatment alone (Table 1).

**Table 1:** *In vitro* cytotoxicity (IC_50_ ng/ml) of ABT-737, ABT-263, or ABT-199 in combination with Mel-14-PE38KDEL immunotoxin.

Xenografts/Cell Lines		Mel-14-PE38KDEL	Mel-14-PE38KDEL+ ABT-737^a^	Mel-14-PE38KDEL+ ABT-263^b^	Mel-14-PE38KDEL+ ABT-199^c^
GBM	D-245 MG	>100	4.00±0.71	>100	85.00±25.98
	D-10-0021 MG	>100	3.83±2.02	>100	>100
	D-08-0326 MG	>100	11.25±9.54	>100	>100
Melanoma	H350	42.50±18.48	0.67±0.31	1.25±1.06	11.00±1.41
	DM440	68.30±14.43	1.67±0.58	13.50±2.12	37.50±3.54
	DM443	>100	28.33±11.55	52.50±10.61	96.00±5.66
Breast Cancer	SUM159	>100	19.50±0.71	64.50±7.78	>100
	SUM159-R113	>100	43.0±2.83	>100	>100
	Hs 578T	>100	>100	>100	>100

Purple: fold of improvement in cytotoxicity (IC_50_ ng/ml) >2. Green: fold of improvement in cytotoxicity (IC_50_ ng/ml) >20. IC_50_ values in all colored blocks were significant when compared to that of the immunotoxin alone group (p <0.05).

^a^ ABT-737 concentration used for GBM and melanoma: 20 μM; for breast cancer: 10 μM.

^b^ ABT-263 concentration used for GBM and breast cancer: 5 μM, for melanoma: 10 μM.

^c^ ABT-199 concentration used for GBMs and melanoma: 10 μM; for breast cancer: 5 μM.

**Table 2:** *In vitro* cytotoxicity (IC_50_ ng/ml) of ABT-737, ABT-263, or ABT-199 in combination with 9.2.27-PE38KDEL immunotoxin.

Xenografts/Cell Lines		9.2.27-PE38KDEL	9.2.27-PE38KDEL+ ABT-737^a^	9.2.27-PE38KDEL+ ABT-263^b^	9.2.27-PE38KDEL+ ABT-199^c^
GBM	D-245 MG	>100	0.078±0.063	>100	6.75±0.35
	D-10-0021 MG	>100	0.045±0.007	9.00±1.41	8.00±2.83
	D-08-0326 MG	>100	0.40±0.14	>100	>100
Melanoma	H350	11.67±2.89	0.21±0.11	0.23±0.11	3.25±1.06
	DM440	>100	0.52±0.17	8.00±1.41	>100
	DM443	>100	1.77±0.87	4.25±1.06	15.00±7.07
Breast Cancer	SUM159	>100	50.00±21.21	>100	>100
	SUM159-R113	>100	5.75±0.35	>100	>100
	Hs 578T	>100	>100	>100	>100

Purple: fold of improvement in cytotoxicity (IC_50_ ng/ml) >2. Green: fold of improvement in cytotoxicity (IC_50_ ng/ml) >20. Blue: fold of improvement in cytotoxicity (IC_50_ ng/ml) >100. Orange: fold of improvement in cytotoxicity (IC_50_ ng/ml) >1000. IC_50_ values in all colored blocks were significant when compared to that of the immunotoxin alone group (p <0.05).

^a^ ABT-737 concentration used for GBM and melanoma: 20 μM; for breast cancer: 10 μM.

^b^ ABT-263 concentration used for GBM and breast cancer: 5 μM, for melanoma: 10 μM.

^c^ ABT-199 concentration used for GBMs and melanoma: 10 μM; for breast cancer: 5 μM.

These data suggest substantial inherent resistance to CSGP4-targeting ITs in the majority of cancer cells expressing the target. To explore the utility of pro-apoptotic enhancers in countering this resistance, we first evaluated the sensitivity of the cell lines in our panel to ABT-737, ABT-263, ABT-199, A-1155463, or S63845 at a concentration of 5–20 μM (see S1, S2A, S2B and S2C Figs for the IC_50_ values). The absorbance of ABT/A-1155463/S63845-treated (Test) vs. vehicle-treated cells (0.5% DMSO in media) (Control) was used to calculate cell viability. A concentration of ABT/A-1155463/S63845 compound that yielded at least 70% cell viability (sub-therapeutic dose) in all three cell lines from GBM, melanoma, or breast cancer was selected for combination therapy. Thus, the concentration of ABT-737 chosen for combination with the ITs was 20 μM (GBM, melanoma), and 10 μM for breast cancer cell lines (S1A, S1B and S1C Fig). For ABT-263 combinations, we chose concentrations of 5 μM (GBMs, breast cancer), and 10 μM for the melanoma cell lines (S1D, S1E and S1F Fig). For ABT-199, the concentrations were 10 μM (GBMs, melanoma), and 5 μM for the breast cancer models (S1G, S1H and S1I Fig). Finally, a concentration of 10 μM (GBM, breast cancer) and 20 μM (melanoma) were chosen for A-1155463 while 5 μM (GBM, breast cancer) and 10 μM were chosen for S63845 (melanoma) S2A, S2B and S2C Fig.

### *In vitro* cytotoxicity of IT and ABT combination therapies

The cytotoxicity (IC_50_) of IT monotherapies or Mel-14-PE38KDEL/9.2.27-PE38KDEL+ABT or 9.2.27-PE38KDEL+A-1155463/S63845 combination therapies are summarized in Tables 1 and 2, and S2D Fig. In all cell lines tested, except for Hs 578T, 9.2.27-PE38KDEL produced stronger synergy with all three ABT compounds than Mel-14-PE38KDEL (Tables 1 and 2). With the 9.2.27-PE38KDEL+A-1155463/S63845 combinations, overall a lower efficacy (DM440-IC_50_ = 8–23 ng/ml and D-10-0021 MG-IC_50_ = 160–200 ng/ml) or no efficacy (SUM159-R113-IC_50_ = >100 ng/ml), compared to the 9.2.27-PE38KDEL+ABT combinations was observed (S2D Fig). Of the three ABT compounds tested, ABT-737 produced the most significant improvement in IC_50_ values with both ITs (Tables 1 and 2). As such, 9.2.27-PE38KDEL+ABT-737 combination induced varying degrees of synergy among the different tumor cell lines (Table 2): for breast cancer models, the 9.2.27-PE38KDEL+ABT-737 combination synergy was most robust in SUM159-R113 (IC_50_ = 5.75 ng/ml), representing a 17.4-fold improvement of cytotoxicity compared to IT monotherapy. Within the melanomas, the 9.2.27-PE38KDEL+ABT-737 combination worked best in DM440 (IC_50_ = 0.52 ng/ml), enhancing the IT monotherapy cytotoxicity by at least 192-fold. The most potent synergy among all 12 tumor cells tested was observed with the GBM xenograft D-10-0021 MG (IC_50_ = 0.045 ng/ml), yielding a >1,000-fold improvement of the IC_50._ Furthermore, Combination Index analysis in D-10-0021 MG showed that even at the lowest IT concentration tested (0.1 pg/ml), the 9.2.27-PE38KDEL+ABT-737 combination demonstrated strong synergistic activity (CI = 0.135) (S1 Table). Moderate synergy (CI = 0.622 to 0.719) was observed in DM440 cells with the 9.2.27-PE38KDEL+ABT-737 combination at concentrations of ≤0.1 ng/ml (S1 Table). For the SUM159-R113 cells, synergy with the combination therapy was observed only at concentrations of ≥10 ng/ml (CI = 0.0172) (S1 Table).

### Basal expression of furin and selected Bcl-2 family proteins in GBM, melanoma, and breast cancer cells

To mechanistically unravel the observed synergistic effects, the basal expression levels of furin, Bcl-2, Bcl-xL, and Mcl-1 were analyzed in our panel of GBM, melanoma, and breast cancer cells (S3 Fig). Compared to both GBM and breast cancer cells, the melanoma cell lines DM440 and DM443 demonstrated higher expression of furin (S3 Fig). Pro-survival Bcl-2 family proteins Bcl-2 and Bcl-xL are targets of both ABT-737 and ABT-263 while ABT-199 exhibits high specificity to Bcl-2 [26]. Expression of Bcl-2 was only detected in DM440. Varying levels of Bcl-xL expression was detected in all cell lines tested while higher expression levels were obvious in SUM159 and DM440 cell lines (S3 Fig). Mcl-1 is a pro-survival Bcl-2 family protein with a short half-life [33]. While Mcl-1 is not a direct target of any of the ABT compounds, its expression level exhausts rapidly upon treatment with agents that induce protein synthesis inhibition, such as the PE38 ITs [33]. Mcl-1 expression was noted in all cell lines tested with higher levels seen in DM440, DM443, and D-10-0021 MG (S3 Fig).

### Assessment of prosurvival and proapoptotic Bcl-2 family proteins following 9.2.27-PE38KDEL/ABT-737 mono or combination therapy

The difference in the susceptibility of D-10-0021 MG, DM440, and SUM159-R113 cells to the 9.2.27-PE38KDEL, ABT combinations impelled us to examine the role of selected prosurvival (Bcl-xL and Mcl-1) and proapoptotic (Bim and Bax) Bcl-2 members in potentiating tumor cell death (S4 Fig). Our analysis indicated no significant difference either in the basal or post-treatment levels of all four Bcl-2 family members in all three cell lines tested. A notable decrease in the levels of all four proteins corresponding with PARP cleavage and induction of apoptosis was observed in D-10-0021 MG, DM440, and SUM159-R113 cells. Thus, 9.2.27-PE38KDEL+ABT-737 combination therapy resulted in protein synthesis inhibition followed by degradation of Bcl-xL, Mcl-1, Bim, and Bax in all three cell lines thereby excluding the participation of these proteins in 9.2.27-PE38KDEL+ABT-737 mediated cell death.

### Internalization of the 9.2.27-PE38KDEL alone or in combination with ABT-737

**Fig 2:**
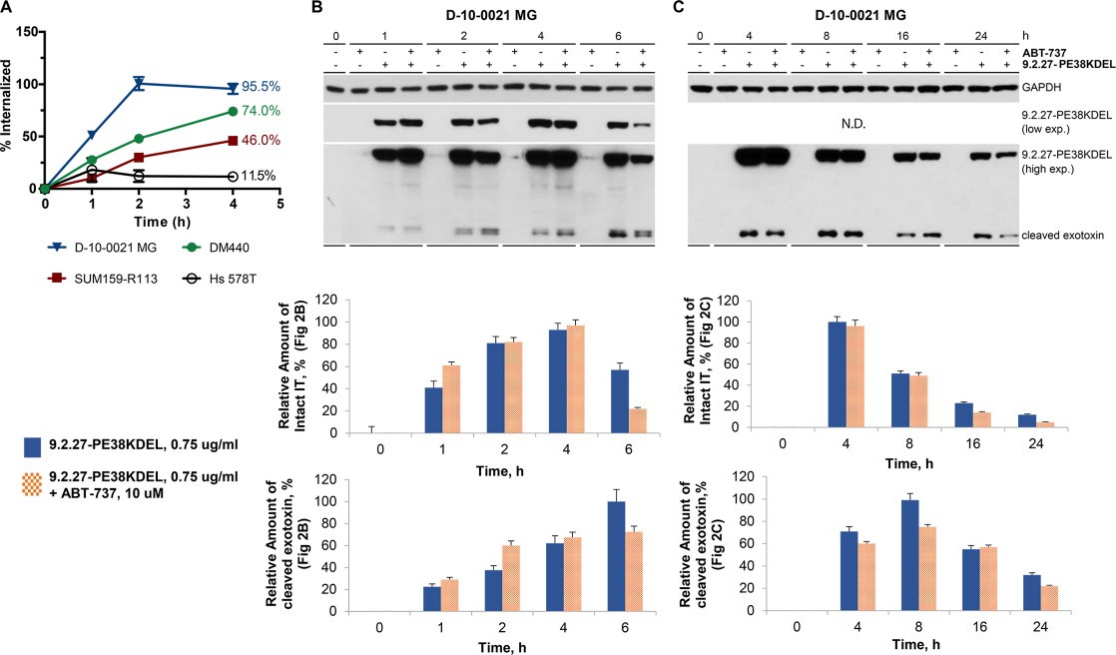
Time course analysis of internalization of 9.2.27-PE38KDEL on selected xenografts and cell lines, by flow cytometry and western blot. **A.** The percentage of IT internalized into the cells at 4 h by flow cytometry was labeled for each xenograft or cell line, in its corresponding color. **B and C.** GBM D-10-0021 MG cells were left untreated or treated with 9.2.27-PE38KDEL (0.75 μM) only, ABT-737 (10 μM) only, or the combination from 1 h to 24 h. Cell lysates were subjected to western blot analysis with GAPDH or *Pseudomonas* exotoxin antibodies. Lower panels represent the relative intracellular accumulation of intact 9.2.27-PE38KDEL and furin processed cleaved exotoxin (PE38KDEL). The values represent the average of 3 experiments, and the error bars represent SEM.

Next we investigated the role of CSPG4 internalization to account for the difference in the sensitivity of tumor cells to the 9.2.27-PE38KDEL, ABT combinations. Flow cytometry analysis revealed that D-10-0021 MG, DM440, and SUM159-R113 cells internalized 95.5%, 74%, and 46% of the surface-bound 9.2.27-PE38KDEL by 4 h, respectively (Fig 2A). 9.2.27-PE38KDEL internalization was the least efficient in Hs 578T cells, with only 11.5% of IT internalized at 4 h (Fig 2A). Internalization of 9.2.27-PE38KDEL was examined by western analysis of D-10-0021 MG cells treated with 9.2.27-PE38KDEL, ABT-737, or the combination at different intervals (Fig 2B). One hour post-treatment, 41% and 61% of intact IT was internalized with the 9.2.27-PE38KDEL and 9.2.27-PE38KDEL+ABT-737 therapies, respectively. The maximal level of intact IT internalization with either monotherapy or the ABT-737 combination was reached at 4 h post-treatment (the relative band intensity of which was arbitrarily set as 100% for comparison). Notably, when compared to IT monotherapy, ABT-737 addition did not significantly affect the maximum level of intracellular, intact IT at 2–4 h post-treatment. An antibody against the ADP-ribosylating catalytic domain of *Pseudomonas* exotoxin was used to determine intact IT internalization and the rate of intracellular IT cleavage by furin. The 38 kDa cleaved IT fragment, was detected 1 h post-treatment with 9.2.27-PE38KDEL alone or in combination with ABT-737 (Fig 2B). A gradual increase in the accumulation of the cleaved exotoxin was observed up to 6 h of treatment. However, only 9–12% of the maximal level of intact, internalized IT was cleaved after 4 h of treatment (Fig 2B).

Since the maximal level of intact 9.2.27-PE38KDEL was detected intracellularly at 4 h post-treatment, we extended the time course study to 24 h for examining the accumulation of intact/cleaved IT (Fig 2C). The results confirmed that for D-10-0021 MG cells treated with either the IT monotherapy or the combination, the intracellular level of intact IT gradually declined after 4 h post treatment. Accumulation of cleaved exotoxin continued to increase till 8 h post-treatment, after which it began to decline, possibly due to proteasomal or/and caspase-dependent degradation (Fig 2C).

### Inhibition of global translation and poly ADP ribose polymerase (PARP) cleavage by mono and combination therapies

**Fig 3:**
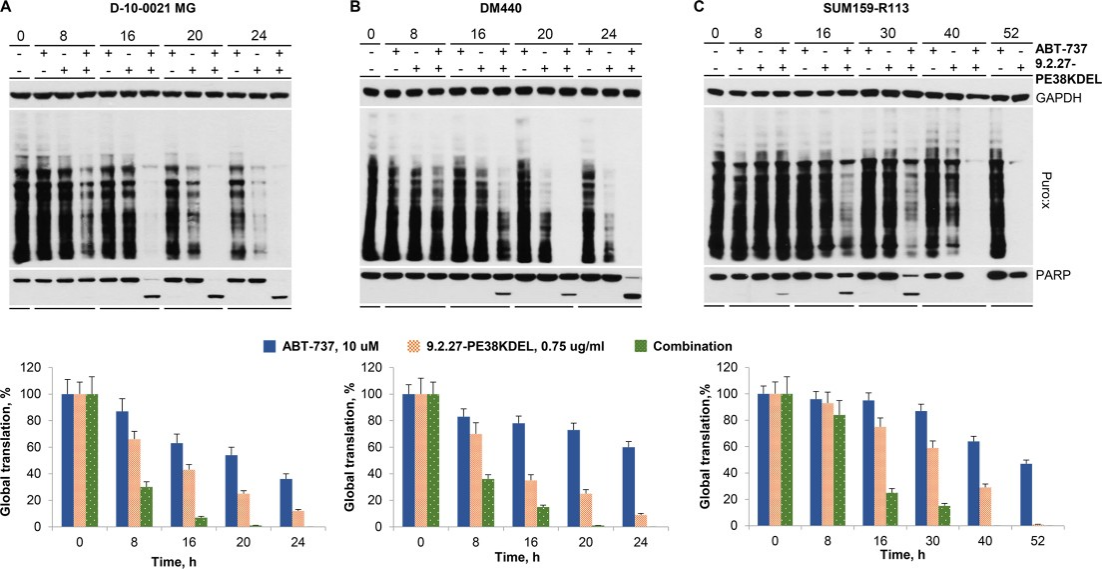
ABT-737 and 9.2.27-PE38KDEL induced changes in global translation and PARP in D-10-0021 MG, DM440, and SUM159-R113 cells. **A-C.** Inhibition of global translation and cleavage of PARP in D-10-0021 MG (A), DM440 (B) and SUM159-R113 (C) at various time points following the treatment of 10 μM ABT-737, 0.75 μg/ml 9.2.27-PE38KDEL, or the combination. During the last 15 min of the incubation interval, the cells were treated with 5 μM puromycin. Upper panels in A-C represent western blot analysis with GAPDH, puromycin, and PARP antibodies. Lower panels in A-C show the time course of global translation. The values represent the average of 3 experiments, and the error bars represent SEM.

Since IT-mediated cytotoxicity is through protein synthesis inhibition followed by apoptosis [11], puromycylation assays were conducted to assess the rate of global protein synthesis inhibition upon IT treatment in D-10-0021 MG, DM440, and SUM159-R113 cells. At 8 h post-treatment of D-10-0021 MG, the 9.2.27-PE38KDEL/ABT-737 combination inhibited global translation by 69%, compared to 36% or 13% with 9.2.27-PE38KDEL and ABT-737 monotherapies, respectively (Fig 3A). Similarly, DM440 (8 h post-treatment) and SUM159-R113 cells (16 h post-treatment), exhibited global protein synthesis reduction to a much greater extent in the combination therapy group compared to either of the monotherapies (Fig 3B and 3C).

Caspase-dependent PARP cleavage, which is often associated with apoptosis, was observed as early as 16 h post combination treatment in D-10-0021 MG, where the majority of intact PARP was cleaved (Fig 3A). On the other hand, neither monotherapy was able to induce detectable PARP cleavage, even at 24 h post-treatment (Fig 3A). Likewise, in both DM440 (Fig 3B) and SUM159-R113 cell lines (Fig 3C), a significantly greater extent of PARP cleavage was observed in the combination therapy compared to the monotherapies. However, near-complete cleavage of PARP (around 90%) was only observed 24 h post combination therapy in DM440 (Fig 3B), and 30 h post combination therapy in SUM159-R113 (Fig 3C). Complete PARP cleavage with the 9.2.27-PE38KDEL/ABT-737 combination at earlier time points in the D-10-0021 MG GBM cells compared to DM440 and SUM159-R113 cell lines, corresponds to elevated *in vitro* cytotoxicity noted with the combination therapy in this model (Table 2). Time course analysis of global translation inhibition and total intact PARP levels (100% intact PARP levels were observed at ≈70–80% translation inhibition) in D-10-0021 MG, DM440, and SUM159-R113 cells confirmed that inhibition of protein synthesis by processed IT resulted in PARP cleavage and apoptosis (S5 Fig).

### Relative amount of cleaved exotoxin following the combination treatment of 9.2.27-PE38KDEL and ABT-737

**Fig 4:**
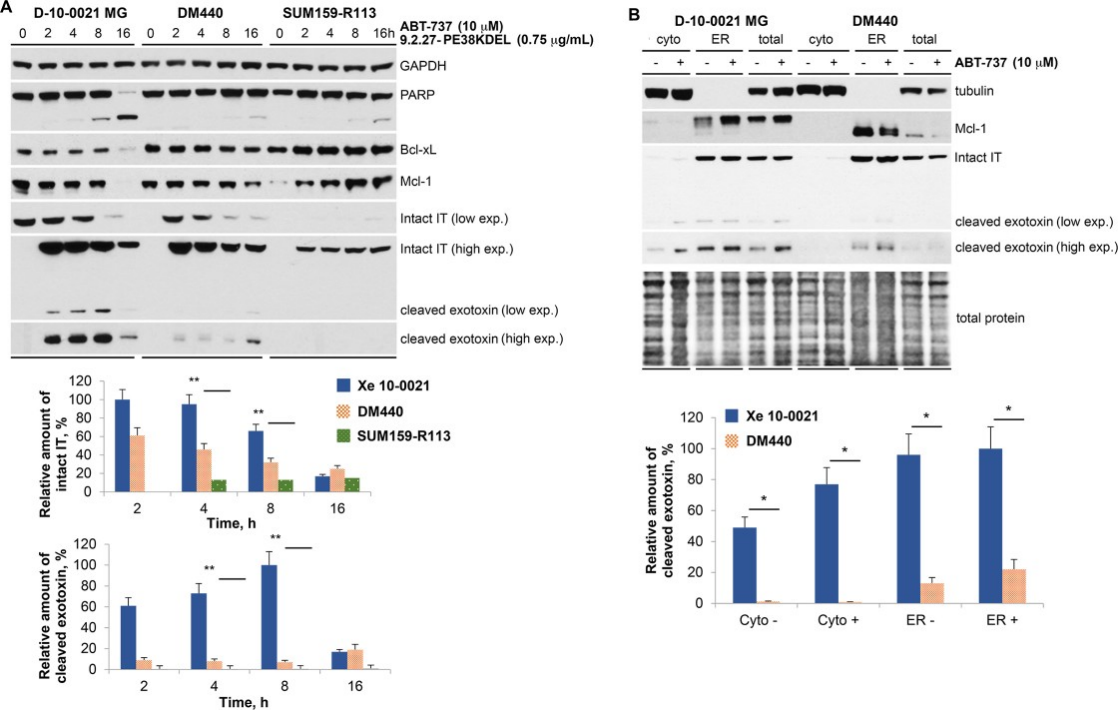
Detection of Bcl-xL, Mcl-1, PARP cleavage, intact IT, and intracellular localization of cleaved exotoxin at indicated time points following the treatment of ABT-737 and 9.2.27-PE38KDEL combination in D-10-0021 MG, DM440, and SUM159-R113. **A.** Expression of Bcl-xL, Mcl-1, cleaved PARP, and cleaved IT at various time points following the combination treatment of 10 μM ABT-737 and 0.75 μg/ml 9.2.27-PE38KDEL in D-10-0021 MG, DM440, and SUM159-R113 cells. The total intracellular extract was used for the analysis. Upper panel represents western blot analysis, and the lower panel displays relative accumulation of furin processed exotoxin in different cell lines. **B.** Distribution of intact IT and cleaved exotoxin in the cytosol, ER, and total lysates of D-10-0021 MG and DM440 cells, at 4 h post treatment of 0.75 μg/ml 9.2.27-PE38KDEL alone or in combination with 10 μM ABT-737. Upper panel represents western blot analysis and the lower panel displays relative localization of cleaved exotoxins in the cytosolic and ER compartments. The values represent the average of 3 experiments, and the error bars represent SEM, and the asterisks indicate significance determined by Student’s t-test (*: p<0.05, **: p<0.01).

Upon receptor binding and internalization, ITs are activated by furin cleavage. To determine IT activation following 9.2.27-PE38KDEL (750 ng/ml) and ABT-737 (10 μM) combination, relative amounts of the intact and cleaved exotoxin were analyzed in D-10-0021 MG, DM440, and SUM159-R113 cells. At 4 h post treatment, D-10-0021 MG demonstrated 2-fold and 7.5-fold more intact intracellular IT than DM440 and SUM159-R113, respectively (Fig 4A). Cleaved exotoxin in D-10-0021 MG cells was detected at 2 h post-treatment and reached maximum levels at 8 h post-treatment. In contrast, significantly lower levels (5–20%) or no cleaved exotoxin was observed in DM440 and SUM159-R113, respectively (Fig 4A). Again, at 16 h post combination therapy, near-complete PARP cleavage was detected in D-10-0021 MG cells, while there was minimal PARP cleavage in both DM440 and SUM159-R113 cell lines (Fig 4A). Bcl-xL levels declined at the 16 h time point in D-10-0021 MG cells, which could be due to caspase-dependent degradation (in correlation with PARP cleavage). No significant change in Bcl-xL levels were observed in DM440 and SUM159-R113 cells (Fig 4A). Total loss of Mcl-1 was noted at 16 h post combination therapy after the appearance of cleaved exotoxin in D-10-0021 MG cells (Fig 4A).

Since furin-processed exotoxin needs to translocate from the ER to cytosol to inhibit protein synthesis, the relative amounts of intact and cleaved exotoxin in the cytosol and ER fractions of D-10-0021 MG and DM440 cells were investigated. Cell lysates were fractionated into cytosolic, ER, and total fractions. Immunoblot analysis of marker protein distributions confirmed the presence of tubulin only in cytosolic fractions, and the ER-membrane protein Mcl-1 only in the ER fraction (Fig 4B), thus establishing proper separation of the different cellular fractions. At 4 h post-treatment for both D-10-0021 MG and DM440 cells, intact ITs were predominantly located in the ER fraction (Fig 4B). In D-10-0021 MG, cleaved exotoxins were detected in both ER and cytosolic fractions, whereas in DM440 cells, they were observed only in the ER fraction (Fig 4B). Our data indicate that both internalization and furin-dependent cleavage of IT was ineffective for SUM159-R113 cell line (Fig 4A). In DM440 cell line, internalization of IT was efficient, but cleavage and translocation of cleaved exotoxin from ER to cytosol was significantly lower than that of D-10-0021 MG cells (Fig 4A). A minimal increase (≈1.6 fold) observed in the cleaved exotoxin in cytosolic fractions of D-10-0021 MG cells following 9.2.27-PE38KDEL+ABT-737 treatment, might contribute to the higher sensitivity of the GBM cells to combination therapy. Thus, in the GBM PDX D-10-0021 MG, rapid internalization (1–2 h) of IT, efficient cleavage (2–8 h) and translocation of cleaved (active) exotoxin from ER to cytosol, complete PARP cleavage, and Bcl-xL and Mcl-1 degradation resulted in robust response to 9.2.27-PE38KDEL+ABT-737 therapy. Taken together the mechanistic studies show that the levels of intracellular IT, processed exotoxin, and PARP cleavage determine the sensitivity of tumor cells to the combination treatment. Our study provides a rationale for the difference in the *in vitro* efficacy of GBM, melanoma, and breast cancer cells to the 9.2.27-PE38KDEL+ABT-737 combination (Table 2).

### *In Vitro* analysis of 9.2.27-PE38KDEL+ABT-737 therapy on CSPG4 mediated signaling pathways

Since CSPG4 activated signaling pathways are known to be involved in tumor cell growth, adhesion, migration, and chemoresistance [34–36] we analyzed the *in vitro* effect of 9.2.27-PE38KDEL/ABT-737 mono or combination therapy on FAK, PKCα, and AKT pathways in D-10-0021 MG, DM440, and SUM159-R113 cells (S6 Fig). An initial increase of p-FAK at earlier time points specifically in 9.2.27-PE38KDEL and 9.2.27-PE38KDEL+ABT-737 therapy groups in D-10-0021 MG and DM440 cells was followed by a decrease at later time points (S6A and S6B Fig). However, these changes were not significant. The levels of p-FAK did not change after treatment in SUM159-R113 cell line (S6C Fig). A significant drop in p-AKT was observed as early as 1 h in D-10-0021 MG GBM cells and by 4–8 h in DM440 and SUM159-R113 cell lines (S6 Fig). We did not observe any change in the levels of p-PKCα or PKCα in D-10-0021 MG and SUM159-R113 cells while the levels were extremely low for detection in DM440 levels (S6 Fig). Our data indicate no difference in the CSPG4 downstream signaling pathways in D-10-0021 MG, DM440, and SUM159-R113 cells post combination therapy.

### *In vivo* efficacy of 9.2.27-PE38KDEL and ABT-737 combination therapy

**Fig 5:**
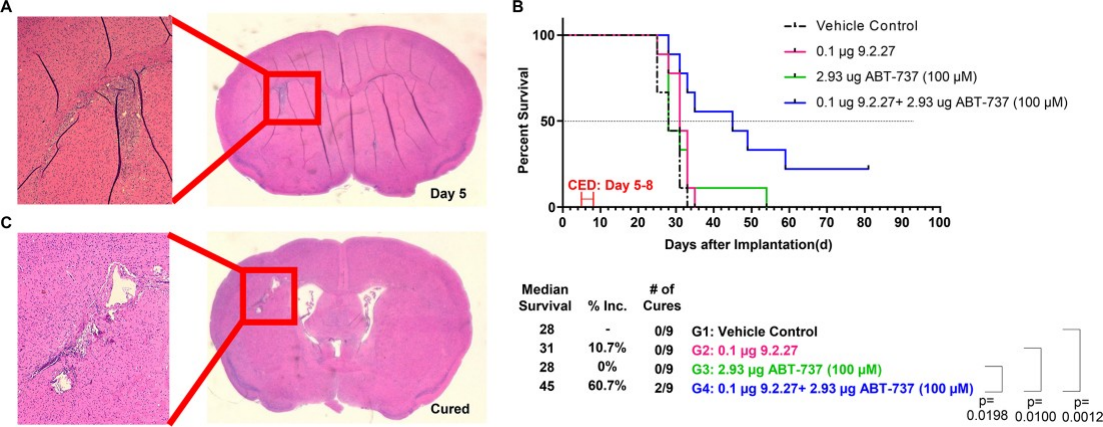
An Intracranial model of GBM xenograft D-10-0021 MG treated with 9.2.27-PE38KDEL, ABT-737, or the combination. **A.** H&E staining of a representative mouse brain section harvested on Day 5 post-tumor implantation. **B.** Survival curve post-therapy and statistical analysis. **C.** H&E staining of a representative brain section harvested from one of the cured mice post 9.2.27-PE38KDEL and ABT-737 combination therapy.

Orthotopic mouse model of primary brain tumors were established using GBM patient-derived xenograft D-10-0021 MG cells. Histological analysis of brains five days post-tumor implantation demonstrated the presence of tumor mass in D-10-0021 MG model (Fig 5A). Thus, day five post-implantation was chosen for treatment initiation. Convection-enhanced delivery (CED), utilizing osmotic pumps, has been successfully used to bypass the blood-brain barrier and to deliver ITs directly into brain tumors [18]. The toxicity of 9.2.27-PE38KDEL was evaluated by CED at a total dose of 0.1 μg or 0.3 μg in mice bearing DM440 intracranial tumors. Toxicity events at an occurrence rate of 1–2 mice per every ten mice were observed only in the 0.3 μg 9.2.27-PE38KDEL group. A total dose of 100 μM (2.93 μg) of ABT-737 by CED was found to be safe for the mice without causing any toxicity. Therefore, a total dose of 0.1 μg of 9.2.27-PE38KDEL and 100 μM of ABT-737 were chosen for the *in vivo* combination studies.

In the D-10-0021 MG model, orthotopic delivery of 100 μM of ABT-737 did not improve MS compared to the vehicle control (p = 0.2871, Fig 5B). The 9.2.27-PE38KDEL (0.1 μg) monotherapy exhibited a modest 10.7% increase in MS compared to the control but failed to reach statistical significance (p = 0.0716). Compared to the vehicle control, the 9.2.27-PE38KDEL+ABT-737 combination therapy prolonged the MS by 60.7% (p = 0.0012). The survival benefit of the combination was also statistically significant when compared to the 9.2.27-PE38KDEL (p = 0.0100) or ABT-737 (p = 0.0198) monotherapies. More importantly, 2/9 (22.2%) mice in the combination therapy group were tumor-free, as confirmed by the H&E staining of their brains (Fig 5C) at the termination of the study (Day 81).

**Fig 6:**
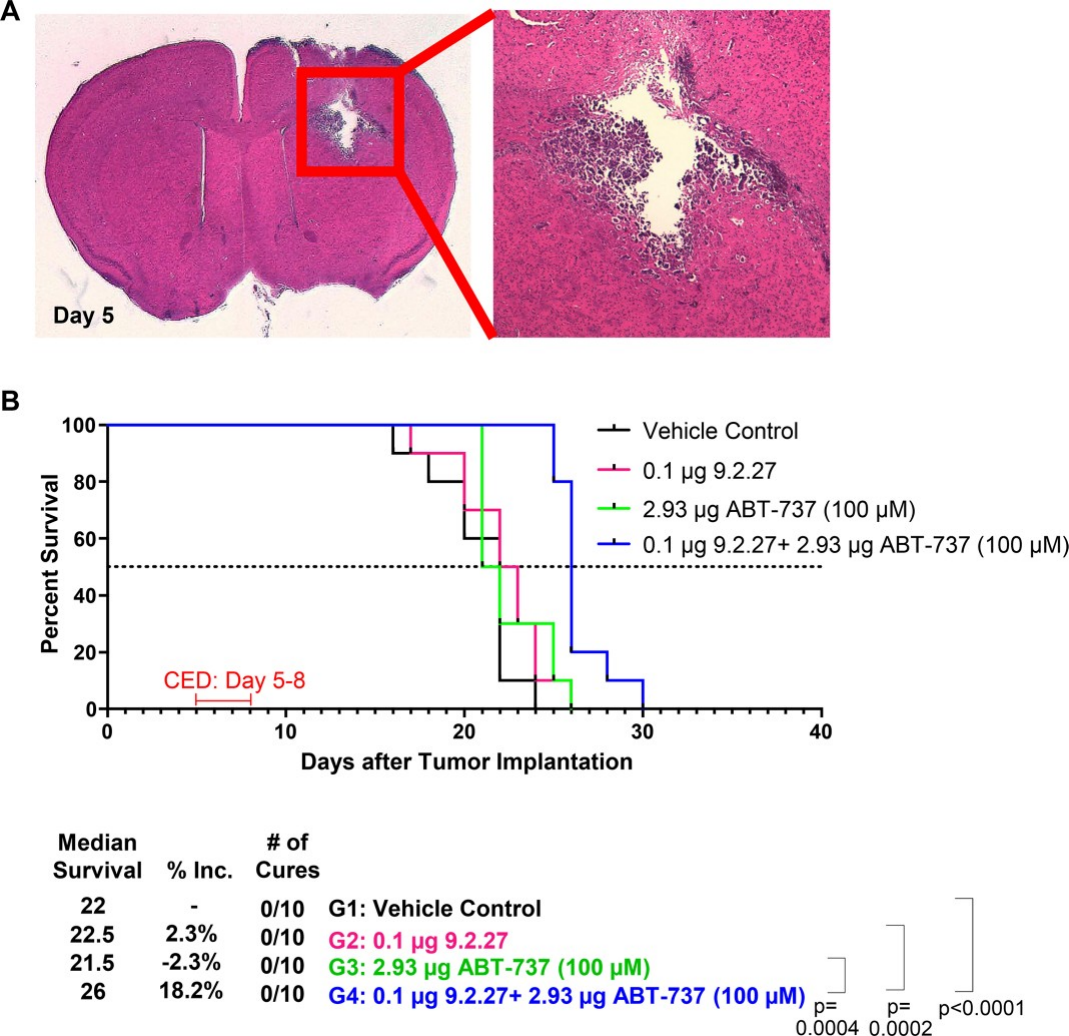
An Intracranial model of melanoma cell line DM440 treated with 9.2.27-PE38KDEL, ABT-737, or the combination. **A.** H&E staining of a representative mouse brain section harvested on Day 5 post-tumor implantation. **B.** Survival curve post-therapy and statistical analysis.

Orthotopic mouse model of metastatic brain tumor was established using DM440 melanoma cells. Histological analysis of DM440 brains (Fig 6A) five days post-tumor implantation demonstrated the presence of tumor mass. In the DM440 model, neither 9.2.27-PE38KDEL (p = 0.1667) nor ABT-737 monotherapies improved MS (p = 0.1822) (Fig 6A). However, compared to the vehicle control, there was an 18.2% increase in MS for the combination group (p<0.0001). The survival benefit with the 9.2.27-PE38KDEL+ABT-737 combination was also statistically significant compared to the ABT-737 (p = 0.0004) or the 9.2.27-PE38KDEL (p = 0.0002) monotherapies.

## Discussion

Brain tumors are composed of highly heterogeneous tumor cell populations that frequently harbor inherent resistance to therapeutic agents, resulting in incomplete eradication of tumor cells and tumor recurrence. Thus, sensitizing tumor cells is critical for improving the efficacy of IT-based therapies. By combining CSPG4 targeting ITs with ABT-737, targeting Bcl-2 family members, we were able to overcome widespread, inherent IT resistance in a panel of GBM, melanoma, and breast cancer cell lines. The IT+ABT-737 combination improved *in vitro* therapeutic efficacy up to >1,000-fold (Tables 1 and 2). The ability of ABT-737 to sensitize cancer cells to 9.2.27-PE38KDEL-mediated cytotoxicity was mechanistically confirmed through western analysis, where the combination therapy, but not the monotherapies, enhanced exotoxin cleavage, induced global translation inhibition, and PARP cleavage. Accordingly, only the combination therapy generated significant improvement in MS, and in some cases even cures, in mouse models of primary or metastatic brain tumors (Figs 5 and 6).

Our *in vitro* studies demonstrated variability in the sensitivity of individual cell lines/xenografts to 9.2.27-PE38KDEL+ABT-737 combination, i.e., cytotoxicity (IC_50_) improvement of >1000-fold (D-10-0021 MG), >200-fold (DM440), and >17-fold (SUM159-R113) (Table 2). Interestingly, while Hs 578T and D-10-0021 MG cells expressed similar levels of CSPG4 (Fig 1), Hs 578T failed to show improvement in cytotoxicity with all of the combinations tested (Tables 1 and 2), demonstrating that the surface CSPG4 density is not a critical determinant of tumor cell sensitivity to combination therapy. Examination of selected prosurvival and proapoptotic Bcl-2 family proteins at basal level and following 9.2.27-PE38KDEL/ABT-737 mono or combination therapy failed to correlate directly with the sensitivity of tumor cells to the combination therapy (S3 and S4 Figs). Our *in vitro* studies also excluded the participation of CSPG4 signaling pathways in sensitizing the tumor cells to the combination therapy (S6 Fig). However, flow cytometry assay (Fig 2A) revealed that the internalization rate of 9.2.27-PE38KDEL is a major determinant of tumor cell sensitivity to combination therapy.

Once internalized, ITs are processed by furin in the endosome and translocated from ER to the cytosol for ADP ribosylation of EF2 and protein synthesis inhibition. Western analysis revealed that at 8 h post combination treatment there was a 12-fold increase in cleaved exotoxin in D-10-0021 MG compared to DM440 (Fig 4A). Moreover, cell fractionation studies revealed that at 4 h post-treatment (9.2.27-PE38KDEL or 9.2.27-PE38KDEL+ABT-737), cleaved exotoxins were found in both cytosolic and ER fractions of D-10-0021 MG cells, while they were detected only in the ER fractions of DM440 (Fig 4B). Thus, increase in cytosolic levels of cleaved exotoxin in D-10-0021 MG compared to DM440 cells corresponds to their enhanced sensitivity to 9.2.27-PE38KDEL and 9.2.27-PE38KDEL+ABT-737 therapies.

In D-10-0021 MG cells, compared to 9.2.27-PE38KDEL monotherapy there was ≈1.6 fold increase in cleaved exotoxin levels post 9.2.27-PE38KDEL+ABT-737 combination (Fig 4B). Thus, in contrast to previous studies [22], our results in the GBM xenograft D-10-0021 MG demonstrate that the addition of ABT-737 does not significantly alter the translocation of cleaved exotoxin from ER to cytosol. However, we observed PARP cleavage in all three tumor models (D-10-0021 MG, DM440, and SUM159-R113) post combination therapy, but not with 9.2.27-PE38KDEL or ABT-737 monotherapies (Fig 3). The efficiency of PARP cleavage correlated well with *in vitro* and *in vivo* efficacy of the combination therapy.

In the D-10-0021 MG model, compared to vehicle control, 9.2.27-PE38KDEL+ABT-737 combination therapy increased MS by 60.7% (p = 0.0012). The efficacy of combination therapy was highly significant when compared to ABT-737 (p = 0.0198) and 9.2.27-PE38KDEL monotherapies (p = 0.0100; Fig 5A). Furthermore, the combination therapy generated cures in 2/9 mice, as verified by histological staining (Fig 5C). Consistent with the lower sensitivity of DM440 cell line to the combination therapy *in vitro*, a modest increase in survival post combination was observed *in vivo*. Collectively, these data confirmed that the 9.2.27-PE38KDEL+ABT-737 combination therapy was able to overcome tumor cell resistance to IT monotherapy *in vivo*, delayed tumor growth, and, in some cases, generated cures.

While several groups have tried to gain mechanistic insights into various immunotoxin+Bcl-2 inhibitor anticancer therapies, only two studies [23, 33] have shown modest antitumor efficacy (improvement in survival by several days without cures), in *in vivo* subcutaneous small cell lung cancer and melanoma tumor models. To achieve this modest improvement in survival, the authors utilized a total of eight doses of 50 mg/kg of ABT-737 + 0.4 mg/kg immunotoxin [23] or a total of two doses of 50 mg/kg of ABT-737 and 0.031 mg/kg immunotoxin [33]. Importantly, in our current intracranial study, we were able to generate 22% cures and improvement in survival utilizing a single dose of 0.147 mg/kg of ABT-737 and 0.005 mg/kg immunotoxin against the aggressive glioblastoma tumor. The ABT-737 and immunotoxin doses in the current study are ≈680–2,700-fold and ≈12–6,400-fold lower, respectively than the previous studies.

In conclusion, our *in vitro* studies, using a panel of GBM, melanoma, and breast cancer cell lines showed that Bcl-2 inhibitor ABT-737 reversed the resistance of tumor cells to IT treatment. We further confirmed the ability of 9.2.27-PE38KDEL+ABT-737 combination to overcome tumor resistance in orthotopic models of brain tumors. Mechanistic studies using 9.2.27-PE38KDEL and ABT-737 mono- and combination therapy revealed that that increased levels of intracellular IT, cleaved exotoxin, and PARP cleavage were determinants of enhanced sensitivity of tumor cells to the combination. Addition of ABT-737 had little effect on the rate of IT internalization but generated a small increase in the translocation of cleaved exotoxin from ER to cytosol. Our study provides insights into employing IT+ABT combinations for overcoming therapy resistance in primary and metastatic brain tumors.

## Supporting information

S1 Checklist NC3Rs ARRIVE guidelines checklist. (PDF)

S1 Fig *In vitro* cytotoxicity of ABT compounds on human tumor cells. **A-I.** Cytotoxicity of ABT-737, ABT-263, or ABT-199 monotherapy on GBM xenografts (A-C), melanoma cell lines (D-F), and breast cancer cell lines (G-I). IC_50_ values were labeled beside each figure.
(TIF)

S2 Fig *In vitro* cytotoxicity of A-1155463 and S63845 on human tumor cells. **A-C.** Cytotoxicity of A-1155463 and S63845 monotherapy on GBM xenograft D-10-0021 MG (A), melanoma cell line DM440 (B), and breast cancer cell line SUM159-R113 (C). IC_50_ values were labeled beside each figure. D. Cytotoxicity of 9.2.27-PE38KDEL and 9.2.27-PE38KDEL+A-1155463/S63845 combinations on D-10-0021 MG, DM440, and SUM159-R113 cells.
(TIF)

S3 Fig Basal level expression of furin and selected Bcl-2 family proteins in melanoma, breast cancer, and GBM cells. The relative level of furin, Bcl-xL, and Mcl-1 in each cell line is presented below the corresponding panel.
(TIF)

S4 Fig ABT-737 and 9.2.27-PE38KDEL induced changes in prosurvival and proapoptotic Bcl-2 family proteins in D-10-0021 MG, DM440, and SUM159-R113 cells. **A-C.** Expression of prosurvival and proapoptotic Bcl-2 family proteins at various time points following the combination treatment of 10 μM ABT-737 and 0.75 μg/ml 9.2.27-PE38KDEL in D-10-0021 MG (A), DM440 (B), and SUM159-R113 (C) cells. Cell lysates were analyzed by western blot with indicated antibodies.
(TIF)

S5 Fig Quantification of ABT-737+9.2.27-PE38KDEL induced changes (relative amounts) in global translation and PARP in D-10-0021 MG, DM440, and SUM159-R113 cells. **A-C.** Inhibition of global translation and intact PARP levels in D-10-0021 MG (A), DM440 (B) and SUM159-R113 (C) at various time points following 10 μM ABT-737+ 0.75 μg/ml 9.2.27-PE38KDEL combination treatment. Data from Fig 3 were quantified. The values represent the average of 3 experiments.
(TIF)

S6 Fig ABT-737 and 9.2.27-PE38KDEL mediated changes in CSPG4 signaling pathways in D-10-0021 MG, DM440, and SUM159-R113 cells. **A-F.** Analysis of CSPG4 activated signaling pathways in D-10-0021 MG (A, D), DM440 (B, E) and SUM159-R113 (C, F) at various time points following the treatment of 10 μM ABT-737, 0.75 μg/ml 9.2.27-PE38KDEL, or the combination. Panels A, B, and C represent western blot analysis with indicated antibodies, and p-AKT/AKT ratios were quantified and averaged between 3 assays (panels D, E, and F, respectively). The error bars represent SEM, and asterisks indicate significance (p<0.05) by Student’s t-test.
(TIF)

S1 Table Combination index (CI) values of ABT-737 and 9.2.27-PE38KDEL combinations on D-10-0021 MG, DM440, and SUM159-R113 cells. (DOCX)

S1 Materials and methods (DOCX)
