# Supplementary figures and images for: Synergistic antitumor effects of 9.2.27-PE38KDEL and ABT-737 in primary and metastatic brain tumors
Source: PLoS One. 2019 Jan 9;14(1):e0210608. doi: 10.1371/journal.pone.0210608 (PMC6326518; doi:10.1371/journal.pone.0210608)

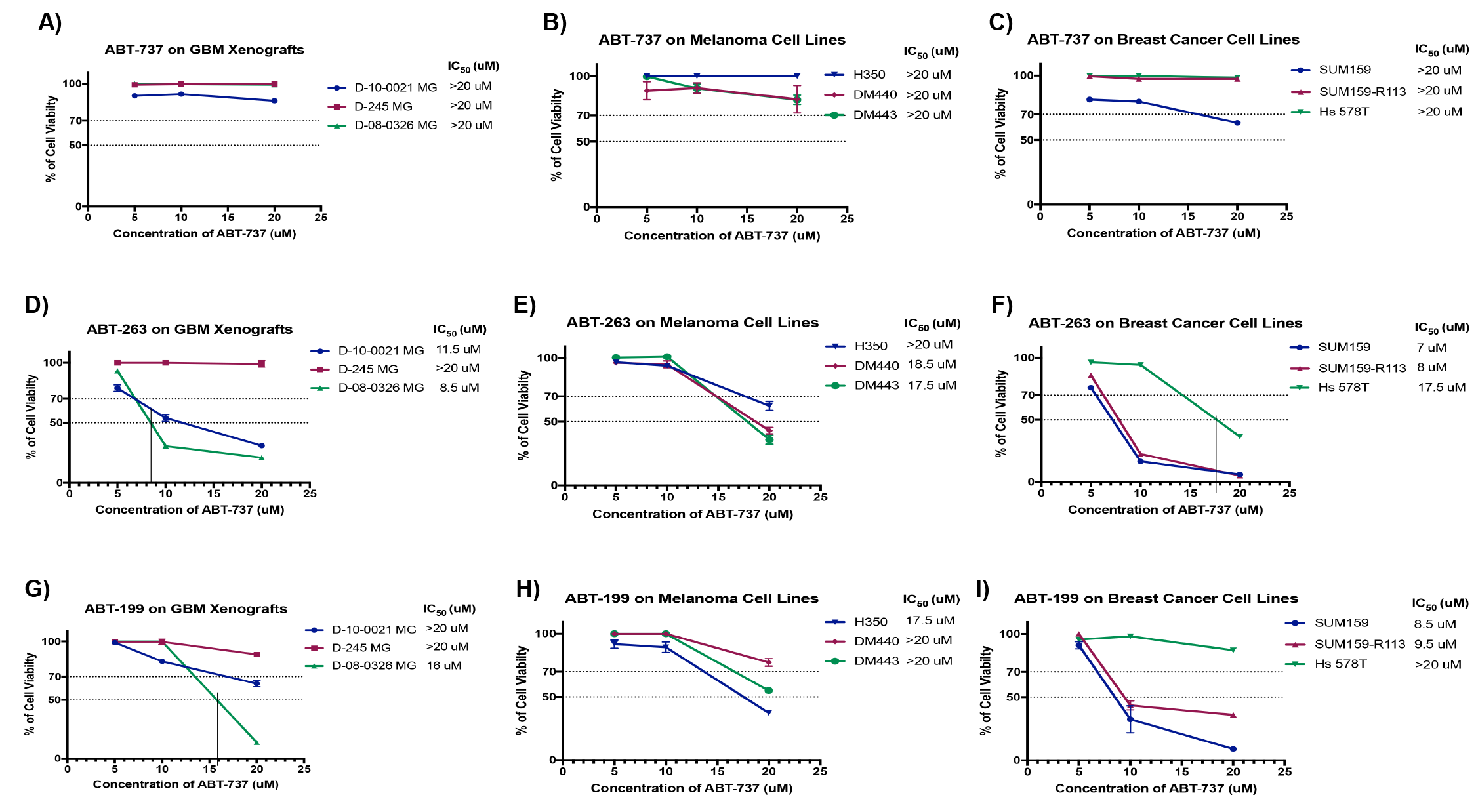

Supplement: S1 Fig — A-I. Cytotoxicity of ABT-737, ABT-263, or ABT-199 monotherapy on GBM xenografts (A-C), melanoma cell lines (D-F), and breast cancer cell lines (G-I). IC50 values were labeled beside each figure. (TIF) [file pone.0210608.s002.tif]

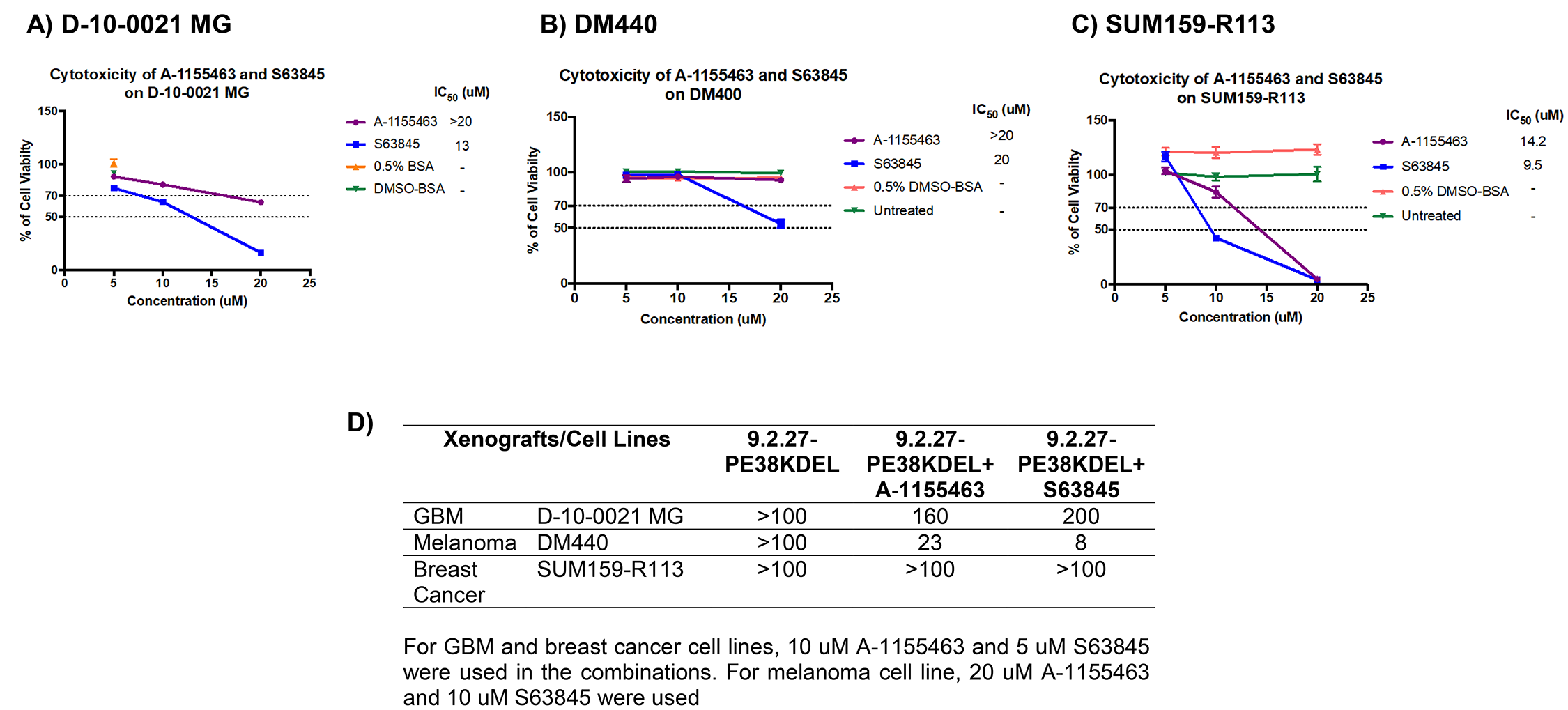

Supplement: S2 Fig — A-C. Cytotoxicity of A-1155463 and S63845 monotherapy on GBM xenograft D-10-0021 MG (A), melanoma cell line DM440 (B), and breast cancer cell line SUM159-R113 (C). IC50 values were labeled beside each figure. D. Cytotoxicity of 9.2.27-PE38KDEL and 9.2.27-PE38KDEL+A-1155463/S63845 combinations on D-10-0021 MG, DM440, and SUM159-R113 cells. (TIF) [file pone.0210608.s003.tif]

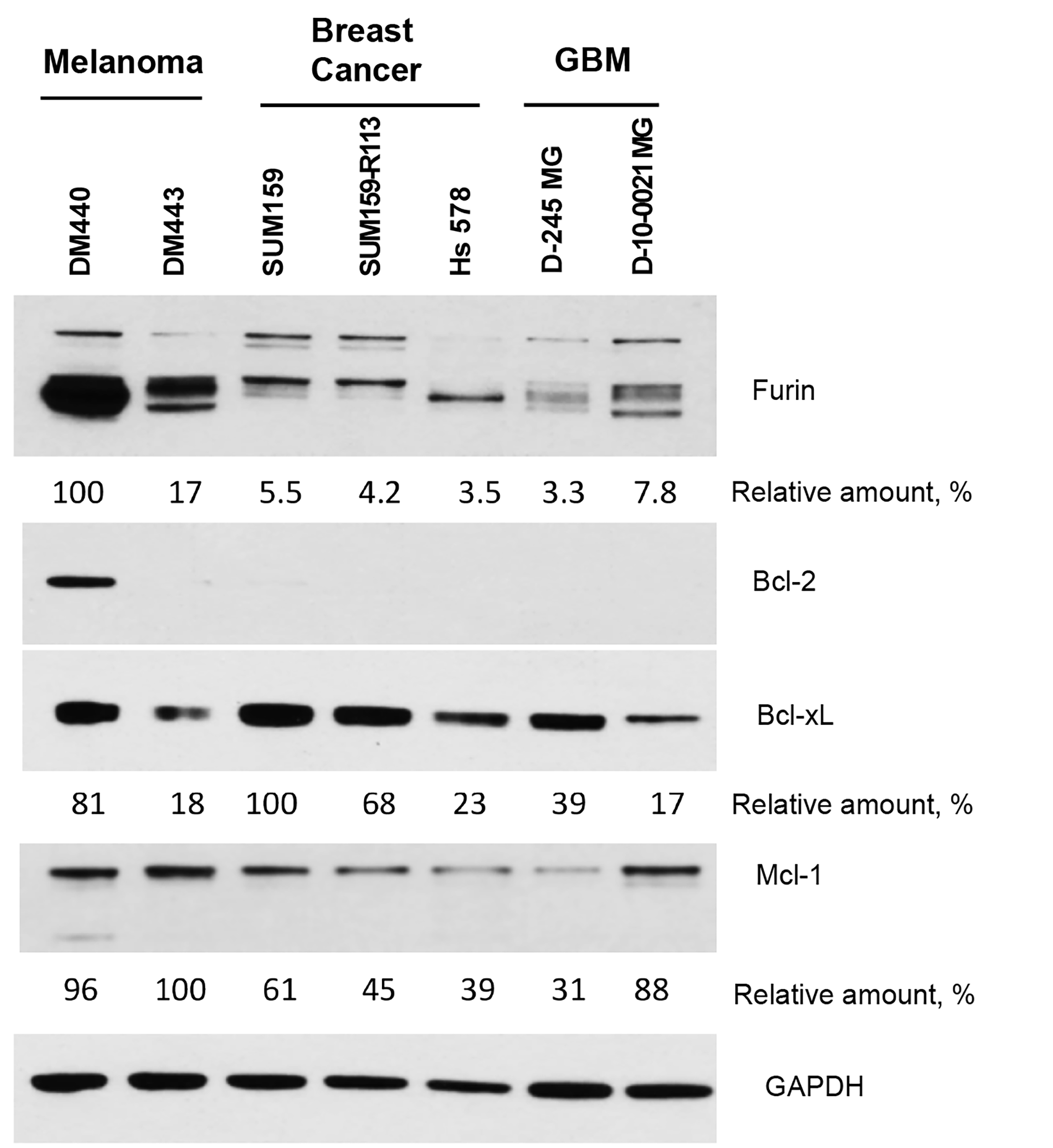

Supplement: S3 Fig — The relative level of furin, Bcl-xL, and Mcl-1 in each cell line is presented below the corresponding panel. (TIF) [file pone.0210608.s004.tif]

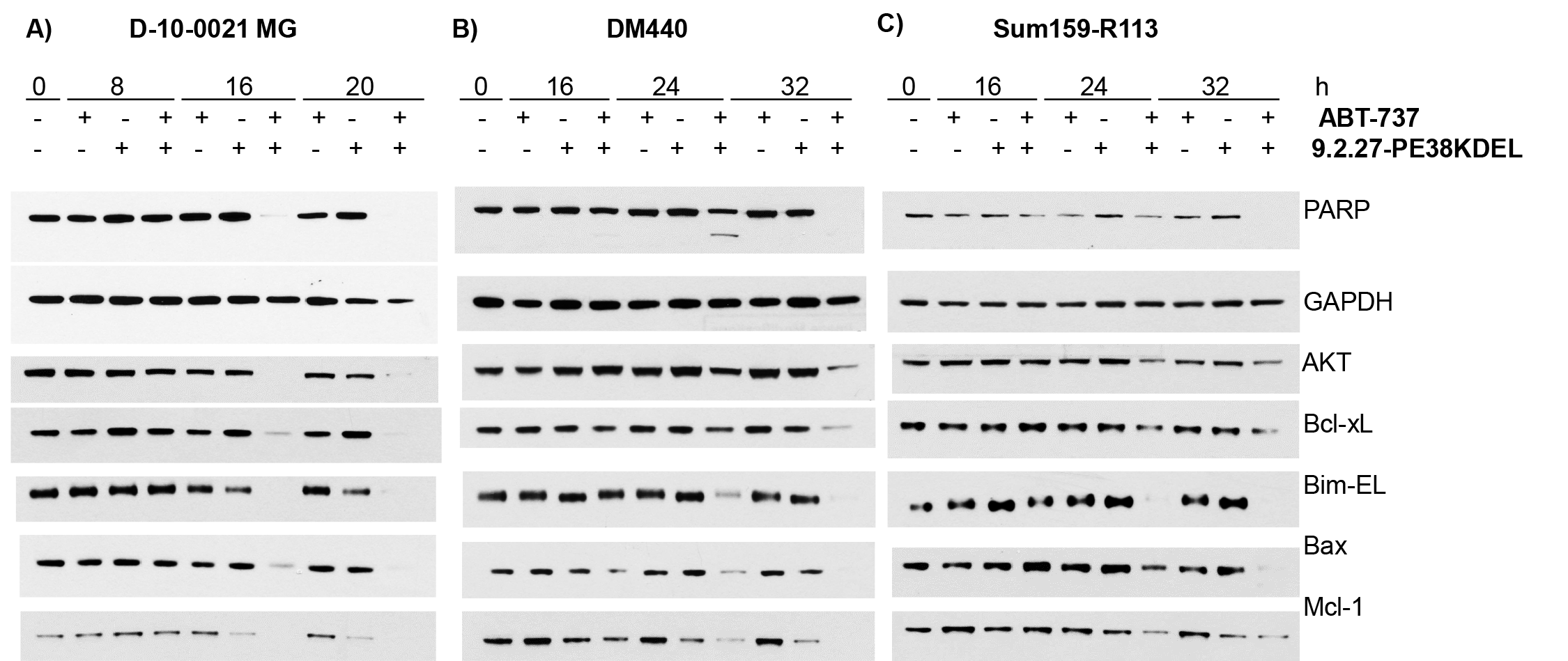

Supplement: S4 Fig — A-C. Expression of prosurvival and proapoptotic Bcl-2 family proteins at various time points following the combination treatment of 10 μM ABT-737 and 0.75 μg/ml 9.2.27-PE38KDEL in D-10-0021 MG (A), DM440 (B), and SUM159-R113 (C) cells. Cell lysates were analyzed by western blot with indicated antibodies. (TIF) [file pone.0210608.s005.tif]

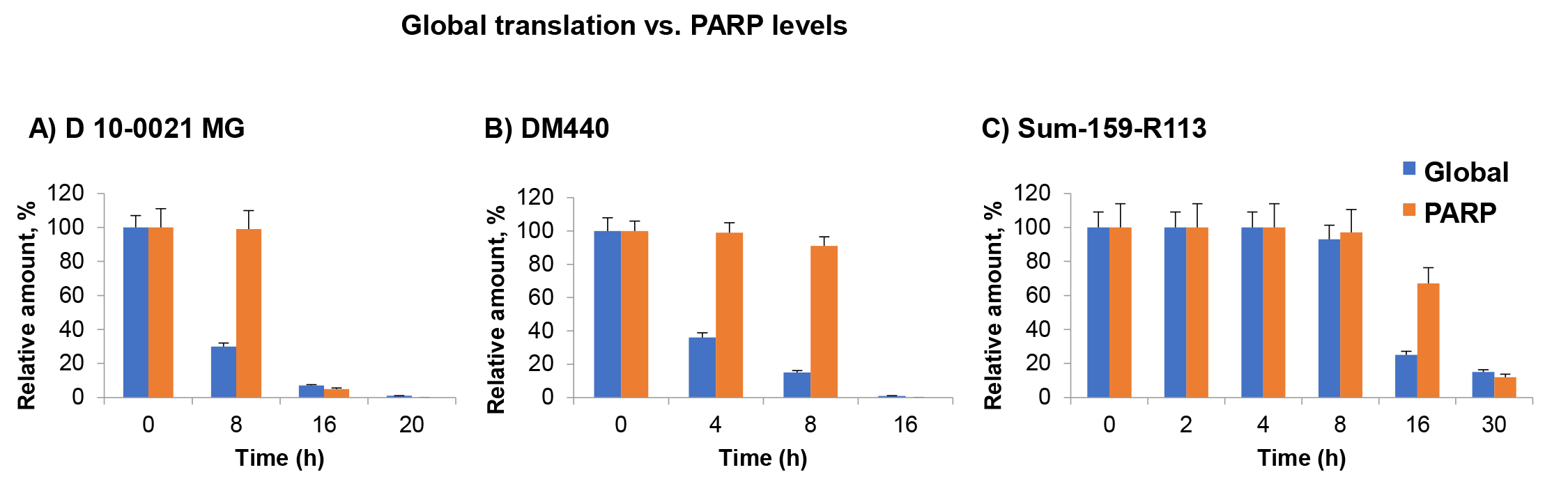

Supplement: S5 Fig — A-C. Inhibition of global translation and intact PARP levels in D-10-0021 MG (A), DM440 (B) and SUM159-R113 (C) at various time points following 10 μM ABT-737+ 0.75 μg/ml 9.2.27-PE38KDEL combination treatment. Data from Fig 3 were quantified. The values represent the average of 3 experiments. (TIF) [file pone.0210608.s006.tif]

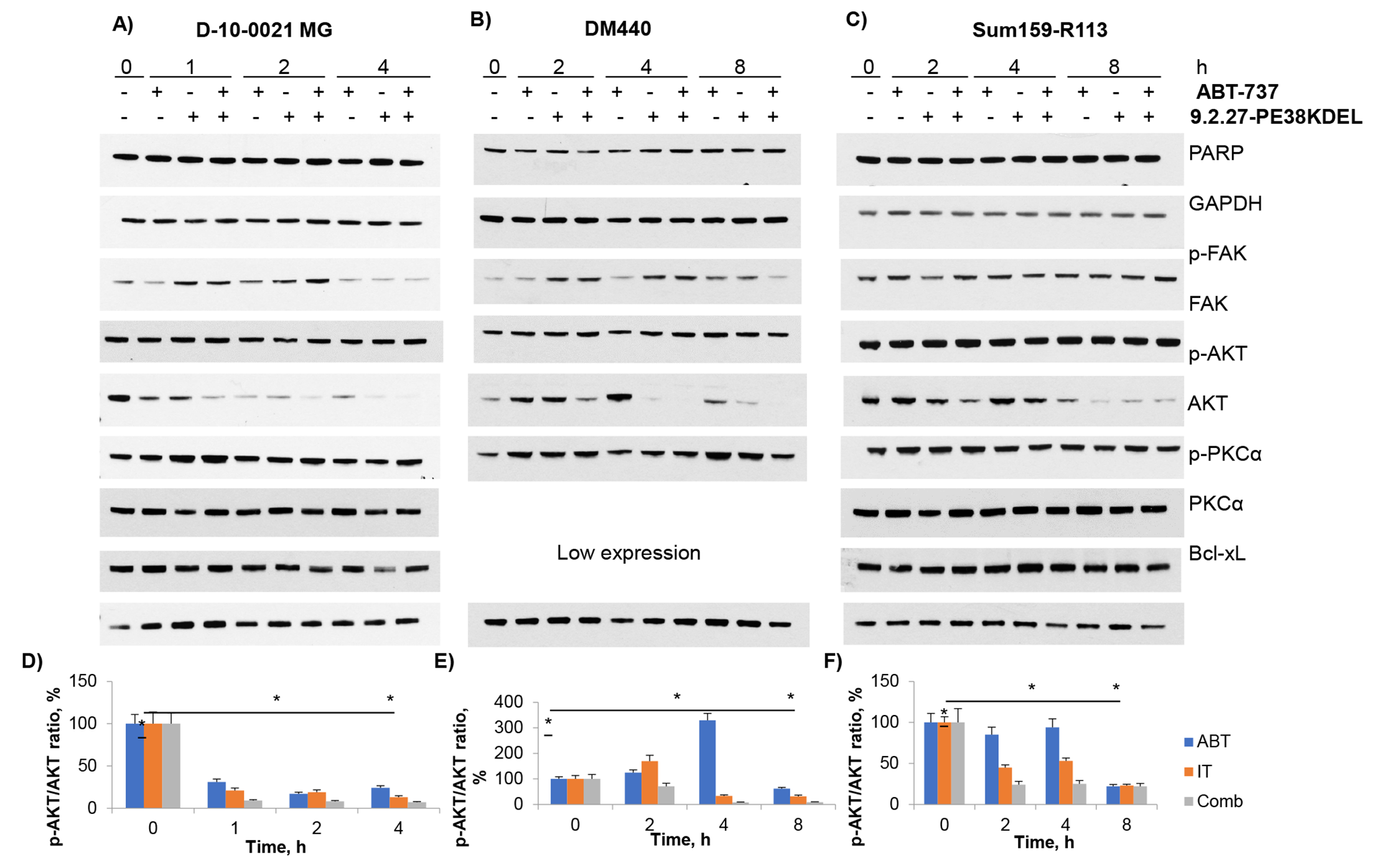

Supplement: S6 Fig — A-F. Analysis of CSPG4 activated signaling pathways in D-10-0021 MG (A, D), DM440 (B, E) and SUM159-R113 (C, F) at various time points following the treatment of 10 μM ABT-737, 0.75 μg/ml 9.2.27-PE38KDEL, or the combination. Panels A, B, and C represent western blot analysis with indicated antibodies, and p-AKT/AKT ratios were quantified and averaged between 3 assays (panels D, E, and F, respectively). The error bars represent SEM, and asterisks indicate significance (p<0.05) by Student’s t-test. (TIF) [file pone.0210608.s007.tif]
