# Supplementary material for: Synergistic antitumor effects of 9.2.27-PE38KDEL and ABT-737 in primary and metastatic brain tumors
Source: PLoS One. 2019 Jan 9;14(1):e0210608. doi: 10.1371/journal.pone.0210608 (PMC6326518; doi:10.1371/journal.pone.0210608)
Supplement: S1 Table — (DOCX) [file pone.0210608.s008.docx]

**Supplementary Table S1: Combination Index (CI) values of ABT-737 and 9.2.27-PE38KDEL combinations on D-10-0021 MG, DM440, and SUM159-R113 cells**

| Concentration of 9.2.27-PE38KDEL (ng/ml) | Combination Index (CI)^1^ | | |
| --- | --- | --- | --- |
|  | **D-10-0021 MG (20 uM ABT-737 + 9.2.27-PE38KDEL)** | **DM440 (20 uM ABT-737 + 9.2.27-PE38KDEL)** | **SUM159-R113 (10 uM ABT-737 + 9.2.27-PE38KDEL)** |
| 100 | 9.71E-08 | 5.40E-04 | 8.68E-03 |
| 10 | 1.20E-07 | 3.25E-04 | 1.72E-02 |
| 1 | 6.29E-07 | 3.11E-03 | NaN^2^ |
| 0.1 | 4.50E-04 | 6.22E-01 | NaN |
| 0.01 | 6.84E-03 | 6.84E-01 | NaN |
| 0.001 | 9.76E-02 | 7.19E-01 | NaN |
| 0.0001 | 1.35E-01 | 6.26E-01 | NaN |

1. CI<1: synergism; CI=1: additive effect; CI>1: antagonism.

2. In cases where neither single nor dual reagents resulted in an inhibitory effect less than 25% of control levels, CI scores were not determined and reported as “not a number” (NaN).
