# Supplementary material for: Synergistic antitumor effects of 9.2.27-PE38KDEL and ABT-737 in primary and metastatic brain tumors
Source: PLoS One. 2019 Jan 9;14(1):e0210608. doi: 10.1371/journal.pone.0210608 (PMC6326518; doi:10.1371/journal.pone.0210608)
Supplement: S1 Materials and methods — (DOCX) [file pone.0210608.s009.docx]

**Supplementary Materials and Methods**

1. Cell Line Authentication:

All cell lines were authenticated periodically by short tandem repeat (STR) profiling. The cell culture supernatant from the melanoma and breast cancer cell lines were tested for mycoplasma infection on 03.08.2013 and 04.21.2016, respectively. All GBM xenograft cells were maintained for 3-4 passages after collection while melanoma and breast cancer cell lines were maintained for 10-12 passages after thawing.

1. Immunotoxins

Development of the Mel-14 and the 9.2.27 IgG2a antibodies have been previously described [1, 2]. The Mel-14-PE38KDEL and 9.2.27-PE38KDEL ITs were constructed by conjugating the Mel-14 scFv and 9.2.27 scFv to the PE38KDEL toxin using a published method [3, 4]. The ITs were expressed as *E Coli* inclusion bodies, refolded, and purified using published methods [3, 4]. The purity of the Mel-14-PE38KDEL and 9.2.27-PE38KDEL ITs was determined to be greater than 90% by sodium dodecyl sulfate-polyacrylamide gel electrophoresis (SDS-PAGE).

1. Preparation of ABT-737, ABT-263, ABT-199, A-1155463, and S63845 for *in vitro* and *in vivo* studies

Small molecule Bcl-2 family inhibitors ABT-737, ABT-263, ABT-199, A-1155463, and S63845 were purchased from Selleck Chemicals (#S1002, #S1001, #S8048, #S7800, and #S8383). For *in vitro* studies, the compounds were dissolved in DMSO at a stock concentration of 10 mM and diluted to working concentrations from 5 μM to 20 μM in the cell culture media, with a final concentration of 0.5% DMSO in the cells. For *in vivo* studies, a working concentration of 100 μM ABT-737 was formulated in a phosphate-buffered saline (PBS)-based vehicle solution containing 5% Captisol and 2% mouse serum albumin (MSA), and fully dissolved using an encapsulation protocol recommended by the manufacturer of Captisol (Ligand Pharmaceuticals). The pH of the solution was adjusted to 7.4 and the osmolality to 280-320 mOsm/Kg H_2_O, a range isosmotic with the cerebrospinal fluid (CSF).

1. Flow cytometry analysis of CSPG4 expression on tumor cells

Dissociation of GBM xenografts, preparation of cells, labeling of Mel-14 IgG antibody, and flow cytometry binding assays were performed as described in an earlier study [5]. To detect the expression of CSPG4, 66.7 nM (equivalent to 10 μg/ml) of AF488-labeled IgG2a isotype control antibody, or the same concentration of the AF488-labeled Mel-14 IgG2a antibody, was used to stain the cells.

1. Q-FACS analysis of CSPG4 antigen density on D-10-0021 MG, DM440, SUM159-R113, and Hs 578T cells

Q-FACS were carried out using Quantum Simply Cellular anti-Mouse IgG beads, according to the manufacturer’s protocol (Bang Laboratories, #815A). The beads were stained with 10 μg/ml of an AF488-labeled IgG2a-AF488 antibody or an equal amount of AF488-labeled Mel-14 IgG antibody. Antibody binding capacity (ABC) was calculated using the lot-specific template provided by the manufacturer.

1. Flow cytometry analysis of internalization of 9.2.27-PE38KDEL on D-10-0021 MG, DM440, SUM159-R113, and Hs 578T cells

Internalization rates of 9.2.27-PE38KDEL on GBM xenograft D-10-0021 MG, melanoma cell line DM440, and breast cancer cells SUM159-R113 and Hs 578T were measured at various time points (0-4 h). Briefly, AF488-labeled 9.2.27-PE38KDEL was then added to 1x10^5^ cells at a protein concentration of 15.63 nM (equivalent to 1000 ng/ml). The reactions were incubated at 4°C for 1 h, washed with 1xPBS, and transferred to a 37°C incubator for different lengths of incubation (0, 1, 2, and 4 h). Then the AF488 fluorescent signals on the cell surfaces were quenched using the AF488 polyclonal antibody (Thermo Fischer Scientific #A-11094). Live cell populations negative for 7-AAD staining (BD #559925) were analyzed for residual fluorescent signals. A vial of reaction kept at 4°C, in the absence of internalization and quenching, was measured as the amount the IT bound to the cell surface at the 0 h time point. The percentage of internalization at different time points was calculated using Equation 1.

$$Percentage of Internalization \left( \% \right)=\frac{Mean AF488 of sample-Mean AF488 of 0h quenched sample}{\begin{aligned} Mean AF488 of 0h nonquenched sample- \\ Mean AF488 of 0h quenched sample \end{aligned}} * 100\%$$

Equation 1

1. WST-1 cytotoxicity assay

The cytotoxicity of Mel-14-PE38KDEL or 9.2.27-PE38KDEL IT on each xenograft, and cell line was determined using the WST-1 assay. Briefly, the cells were seeded in 96-well plates at 1x10^5^ cells/well in triplicates, and cultured overnight. The next day, Mel-14-PE38KDEL or 9.2.27-PE38KDEL IT were serially diluted from 0.01 pg/ml to 100 ng/ml and added to the corresponding triplicate wells. The plates were then incubated at 37°C for 48 hours and the cell proliferation reagent WST-1 (Sigma #5015944001) was then added and incubated with the cells for another 4 hours at 37°C. The plates were analyzed using a plate reader at a measurement wavelength of 440 nm and a reference wavelength of 650 nm. The results were plotted by GraphPad Prism (GraphPad Software). The cytotoxic activity was defined by IC_50_, which is the concentration (ng/ml) of the IT required to inhibit the growth of the cancer cells by 50%.

The cytotoxicity of ABT-737, ABT-263, or ABT-199 on each xenograft and cell line was determined using a similar method as detailed above. The stock ABT solutions in DMSO were serially diluted so that the working concentrations of the ABTs ranged from 5 μM to 20 μM in 0.5% DMSO in the cells. All negative control wells had the same concentration of DMSO without the ABTs. The cytotoxicity of the IT+ABT combinations was determined by treating the cells with serially diluted Mel-14-PE38KDEL or 9.2.27-PE38KDEL (0.0001 ng/ml to 100 ng/ml) in combination with one of the ABT compounds at a fixed concentration. Combination index (CI) values were established using Chou-Talalay method as described.

Reference

1. Carrel S, Schreyer M, Schmidt-Kessen A, Mach JP. Reactivity spectrum of 30 monoclonal antimelanoma antibodies to a panel of 28 melanoma and control cell lines. Hybridoma. 1982;1(4):387-97. Epub 1982/01/01. doi: 10.1089/hyb.1.1982.1.387. PubMed PMID: 6208135.

2. Morgan AC, Jr., Galloway DR, Reisfeld RA. Production and characterization of monoclonal antibody to a melanoma specific glycoprotein. Hybridoma. 1981;1(1):27-36. Epub 1981/01/01. doi: 10.1089/hyb.1.1981.1.27. PubMed PMID: 6208119.

3. Buchner J, Pastan I, Brinkmann U. A method for increasing the yield of properly folded recombinant fusion proteins: single-chain immunotoxins from renaturation of bacterial inclusion bodies. Anal Biochem. 1992;205(2):263-70. Epub 1992/09/01. PubMed PMID: 1332541.

4. Chandramohan V, Bao X, Keir ST, Pegram CN, Szafranski SE, Piao H, et al. Construction of an immunotoxin, D2C7-(scdsFv)-PE38KDEL, targeting EGFRwt and EGFRvIII for brain tumor therapy. Clin Cancer Res. 2013;19(17):4717-27. doi: 10.1158/1078-0432.CCR-12-3891. PubMed PMID: 23857604; PubMed Central PMCID: PMCPMC3766439.

5. Yu X, Pegram CN, Bigner DD, Chandramohan V. Development and validation of a cell-based fluorescent method for measuring antibody affinity. J Immunol Methods. 2017;442:49-53. Epub 2016/12/28. doi: 10.1016/j.jim.2016.12.004. PubMed PMID: 28024998; PubMed Central PMCID: PMCPMC5293658.
